# Dimensional crossover and incipient quantum size effects in superconducting niobium nanofilms

**DOI:** 10.1038/s41598-018-22983-6

**Published:** 2018-03-16

**Authors:** Nicola Pinto, S. Javad Rezvani, Andrea Perali, Luca Flammia, Milorad V. Milošević, Matteo Fretto, Cristina Cassiago, Natascia De Leo

**Affiliations:** 1 School of Science and Technology, Physics Division, University of Camerino, via Madonna delle Carceri 9, Camerino, 62032 Italy; 2 INFN, Sezione di Perugia, via Pascoli, Perugia, 06123 Italy; 3 Istituto Nazionale di Ricerca Metrologica (INRiM), Strada delle Cacce, 91, Torino, 10135 Italy; 4 School of Pharmacy, Physics Unit, University of Camerino, Camerino, 62032 Italy; 5 Department of Physics, University of Antwerp, Groenenborgerlaan 171, B-2020 Antwerp, Belgium

## Abstract

Superconducting and normal state properties of Niobium nanofilms have been systematically investigated as a function of film thickness, on different substrates. The width of the superconducting-to-normal transition for all films is remarkably narrow, confirming their high quality. The superconducting critical current density exhibits a pronounced maximum for thickness around 25 nm, marking the 3D-to-2D crossover. The magnetic penetration depth shows a sizeable enhancement for the thinnest films. Additional amplification effects of the superconducting properties have been obtained with sapphire substrates or squeezing the lateral size of the nanofilms. For thickness close to 20 nm we measured a doubled perpendicular critical magnetic field compared to its large thickness value, indicating shortening of the correlation length and the formation of small Cooper pairs. Our data analysis indicates an exciting interplay between quantum-size and proximity effects together with strong-coupling effects and the importance of disorder in the thinnest films, placing these nanofilms close to the BCS-BEC crossover regime.

## Introduction

Superconducting niobium thin films are being currently investigated for both fundamental and technological applications. Thin films of Nb, grown on suitable substrates, are needed for technological quantum devices, such as Josephson junctions, nano Superconducting QUantum Interference Devices (SQUIDs)^1^, mixers^2^ and single photon detectors^3^. The fabrication and characterization of high quality Nb nanofilms with optimized superconducting properties is therefore of key importance. These layers can be the ideal platform to design nanostructures with reduced dimensionality, such as single or multiple superconducting nanostripes, to control and enhance superconductivity^4–7^, to generate multigap and resonant phenomena^8–13^, to fabricate heterostructures of superconductors with other materials^14^, etc. A plethora of these and other novel nanostructures can be fabricated starting from Nb nanofilms, using readily available techniques of, e.g., electron beam lithography and focused ion beam nano-sculpting^15^.

In this work we report an extended experimental investigation of the superconducting to normal state transition in Nb nanofilms, with a thickness in the range 9 nm to 90 nm. We have measured and derived the main microscopic parameters characterising the normal and the superconducting state, as the mean free path of charge carriers, the critical current, the magnetic penetration depth and the correlation length. Their values are analysed and discussed in terms of the recent theories. There is some evidence of limitations in the existing models. We have explored the important role of the substrate, depositing films on silicon dioxide (SiO_2_) and sapphire (Al_2_O_3_). These form quite different interfaces with Nb, in terms of oxidation at the interface of the grown nanofilms. Finally, we study the effect of lateral dimension of Nb nanofilms with a view to fabricate superconducting nanostripes in the future.

We find a 3D to 2D dimensional crossover in the superconducting properties of the Nb nanofilms as the thickness is reduced below ≈25 nm. We see evidence of the dependence of the critical temperature on thickness related to the interplay between quantum size and proximity effects at the substrate interface. In addition, we provide evidence for Cooper-pair shrinking when approaching the ultra thin regime of film thicknesses, that is discussed within the framework of the Bardeen-Cooper-Schrieffer (BCS) - Bose-Einstein Condensation (BEC) crossover theory^16^. We demonstrate that the thickness dependence of the mean free path causes the Cooper pair shrinking. This result indicates the non trivial interplay between disorder and BCS-BEC crossover. This interplay is known on the BCS weak coupling side of the BCS-BEC crossover, where disorder increases the superconducting critical temperature and amplifies the superconducting fluctuations^17^. Our experimental and theoretical analysis clearly shows that superconducting Nb nanofilms, with thickness between 20 nm and 30 nm, give optimal performance. From a technological point of view, these outcomes fill the gap of information on Nb films, between the atomistic and the mesoscopic range of thickness. They will be useful to guide the fabrication of optimized nanodevices and other superconducting circuitry.

## Results

### Resistivity and critical temperature

**Figure 1:**
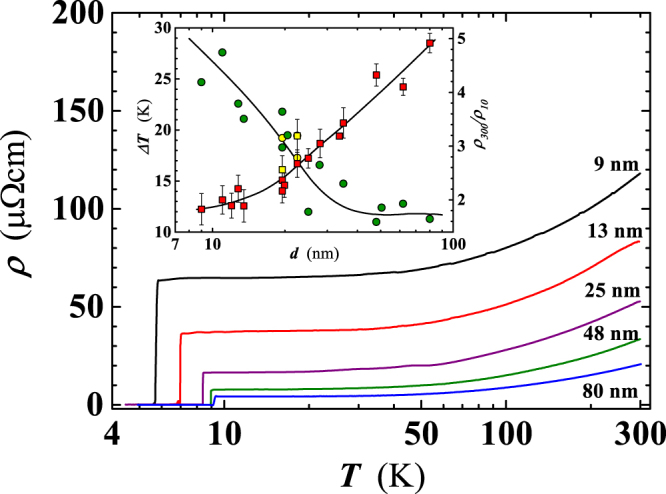
Temperature dependence of the resistivity for selected Nb films, with a thickness ranging from 9 nm to 80 nm. Inset: width of the plateau region (Δ*T*), above *T*_*C*_, as a function of *d* (circles). As Δ*T* we have considered the *T* interval corresponding to a film resistivity change of ±2.5% of *ρ*(*T*) @ 10 K. Squares: resistivity ratio at *T* = 300 K and at *T* = 10 K as a function of *d*. All films have been deposited on SiO_2_ substrates except those, shown by yellow filled symbols, deposited on sapphire.

The temperature dependence of the resistivity, *ρ*(*T*), of Nb films has been investigated as a function of the thickness, *d*, in the range $9\lesssim d\lesssim 80$ nm (see Table 1). For details on fabrication of the films, we refer the reader to the Methods section. The *ρ*(*T*) curves shift upward, while the superconducting transition temperature, *T*_*C*_, decreases as *d* is progressively reduced (Fig. 1). We have found that for films with 9 < *d* < 25 nm, the shift of the *ρ*(*T*) curves is larger than for 25 ≤ *d* < 80 nm (Fig. 1). The 80 nm thick film (#6), being least resistive of those measured, exhibits a room temperature (R.T.) value of *ρ*(*T*) ≈ 20 *μ*Ω cm, approaching the Nb bulk value (15 *μ*Ω cm)^18,19^. Lowering of the resistivity with thicker *d* suggests a gradual reduction of the film defectivity, in accordance with the behavior reported for Nb films by other groups^20–22^. For ${T}_{C} < T\lesssim 45$ K, the *ρ*(*T*) shows a plateau, due to the residual defects in the nanofilm (the nature and the influence of which will be discussed later). Starting from the thinnest films, this plateau rapidly shrinks with increasing *d* up to 25 nm and then attains a nearly constant value for 25 < *d* < 80 nm (see inset of Fig. 1).

**Table 1:** The experimentally measured properties of different Nb nanofilms.

Sample	Thickness (nm)	width (*μ*m)	*T*_*C*_ (K)	Δ*T*_*C*_ (mK)	*ρ*_300_ (*μ*Ω cm)	*RRR* (*ρ*_300_/*ρ*_10_)	${{\boldsymbol{\mu }}}_{{\bf{0}}}{{\boldsymbol{H}}}_{{\boldsymbol{C}}\mathrm{20}{\boldsymbol{\perp }}}$ (T)	*ξ*(0) (nm)
#9	9	50	6.462	50	118.2	1.827	—	—
#17	11	50	6.53	65	97.8	2.00	—	—
#12	12	50	6.475	30	135.7	1.893	—	—
#16	13	50	6.84	19	84.9	2.208	—	—
#H15	13.5	10	6.631	79	70.19	1.889	—	—
#H14S	13.5	10	7.313	75	—	—	—	—
#H1S	19.5	10	7.91	35	31.0	2.563	—	—
#H1	19.5	10	7.72	55	20.7	2.367	4.4 ± 0.10	7.77 ± 0.14
#H5	19.5	10	7.48	49	35.8	2.165	—	—
#H2	20.5	10	7.830	42	42.3	2.272	—	—
#H11S	22.5	10	8.108	28	28.4	3.191	—	—
#H9	22.5	10	7.888	44	30.1	2.675	—	—
#7	25	50	8.399	15	25.6	2.774	—	—
#H13	28	10	8.51	28	25.8	3.049	—	—
#H7	28	10	7.967	65	30.4	2.522	—	—
#H8	33	10	8.505	40	36.3	2.524	2.30 ± 0.03	11.3 ± 0.20
#3	34	50	8.585	16	21.1	3.186	—	—
#H6	35	10	8.519	29	20.9	3.432	—	—
#4	48	10	8.970	16	67.2	4.324	—	—
#H3	50	10	8.630	20	21.1	3.074	—	—
#5	62	50	8.927	10	57.9	4.103	—	—
#6	80	50	9.133	11	20.7	4.917	—	—
#H12	92	10	—	—	—	—	2.12 ± 0.016	11.5 ± 0.10

From left to right: Sample name (an S, following the number, indicates deposition on a sapphire substrate); Film thickness; width of the sample used for the electrical characterisation; *T*_*C*_; Superconducting transition width, Δ*T*_*C*_; Resistivity at 300 K; Resistivity ratio (RR) at 300 K and 10 K; Perpendicular critical magnetic field at 0 K; coherence length in the film plane at 0 K. For resistivity measurements, 1 *μ*A of current intensity has been sourced to all films but #7 (50 *μ*A); #5 (10 *μ*A and 50 *μ*A) and #6 (50 *μ*A).

**Figure 2:**
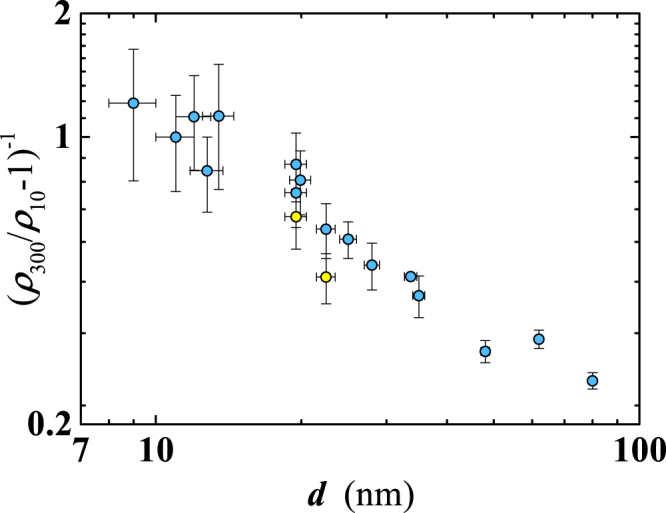
Thickness dependence of the quantity *C* = (*ρ*_300_/*ρ*_10_ − 1)^−1^. *ρ*_300_/*ρ*_10_ is the residual resistivity ratio (i.e. *RRR*). All films have been deposited on SiO_2_ substrates except for those indicated by yellow filled circles, deposited on sapphire.

The gradual improvement of the film quality for larger *d* is also deduced by the thickness dependence both of the residual resistivity ratio, *RRR*, here defined as the *ρ*(*T*) ratio at *T* = 300 K and *T* = 10 K, i.e. *RRR* = *ρ*_300_/*ρ*_10_^23^ and the quantity *C* = (*RRR*−1)^−1^, proportional to defects density in the film^21^. *RRR* grows monotonically with *d*, from ~2, for *d* = 9 ÷ 13 nm, to ~5 at *d* = 80 nm (Fig. 1: inset). These values are comparable with the data reported by Lotnyk *et al*.^23^ and, for *d* < 40 nm, also with the data of Mayadas *et al*.^24^. Agreement with findings of Delacour *et al*.^21^ occurs at the lowest thickness (*d* ≈ 10 nm). Finally, the rapid lowering of *C* with increasing film thickness indicates reduction of the defect density (Fig. 2), in agreement with findings of ref.^21^. It is worth noting that *C* becomes lower, for the same thickness, in those films deposited on the sapphire substrate (see Fig. 2). Possible effects of the substrate on other measured film properties will be addressed later.

**Figure 3:**
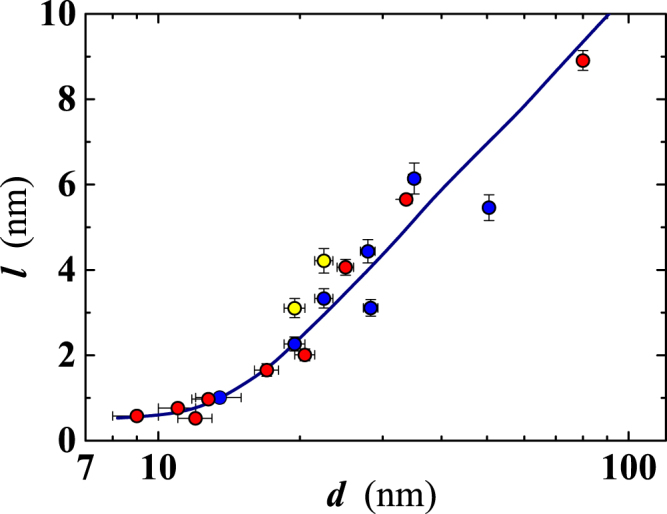
Thickness dependence of the mean free path at 10 K. Data have been derived from the resistivity values measured at the same temperature (i.e. *ρ*_10_) for either 10 *μ*m (blue dots) or 50 *μ*m (red dots) wide films. Yellow filled symbols correspond to films (10 *μ*m wide) deposited on a sapphire substrate; all the other films have been deposited on SiO_2_. Continuous line is a guide for eyes.

Residual resistivity (i.e. *ρ*_10_) can be used to estimate the charge carriers mean-free path, *l*, at *T* = 10 K, assuming the constancy of the product *ρl* = 3.75 × 10^−6^ *μ*Ω cm^2^, *ρ* being the bulk Nb resistivity^25^. Values for *l* range from ≈1 nm, at the lower thicknesses, to ~9 nm at *d* = 80 nm (Fig. 3). Films deposited on sapphire evidence higher *l* values, in agreement with an expected lower defect density in the film matrix (Fig. 3). Later in our analysis, the thickness dependence of *l* will be used to extract the thickness dependence of superconducting coherence length and magnetic penetration depth.

**Figure 4:**
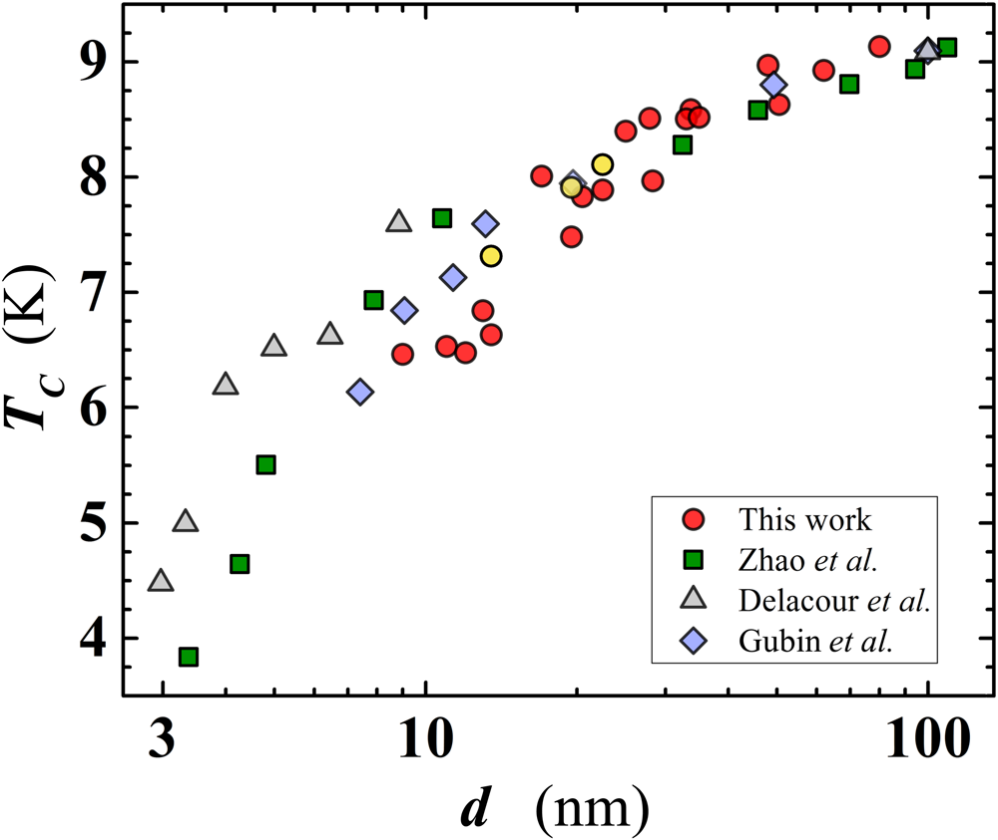
Thickness dependence of the superconducting transition temperature. Circles: data of the present work, for layers deposited on SiO_2_ (red filled) and on sapphire (yellow filled). For comparison, we have added data from: Zhao *et al*. (squares)^20^; Gubin *et al*. (diamonds)^22^; Delacour *et al*. (triangles)^21^.

**Figure 5:**
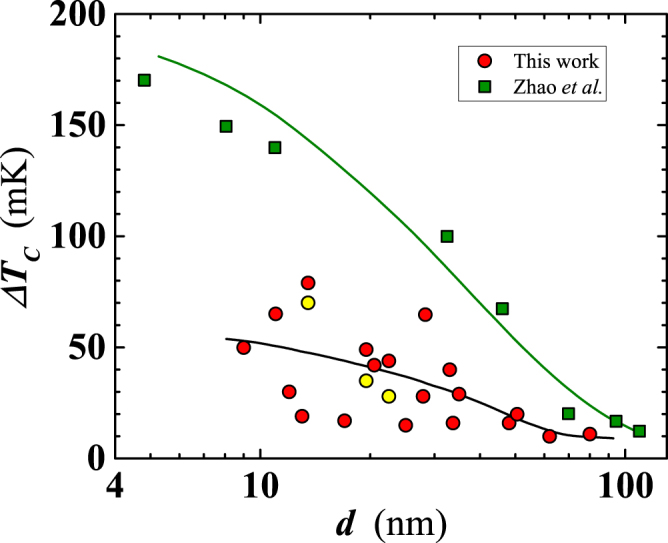
Thickness dependence of the superconducting transition width. Circles: data of the present work, measured in films deposited on SiO_2_ (red filled) and on sapphire (yellow filled). Squares: data taken from Zhao *et al*.^20^. Solid lines serve as a guide for the eyes.

Next, we have studied in detail the dependence of the superconducting transition temperature on film thickness. We have determined *T*_*C*_ as the midpoint between temperatures corresponding to the 10% and the 90% of the normal resistance value, located on the residual resistance plateau of the *R*(*T*) curve, for *T* > *T*_*C*_. The difference of those two *T* values was taken as the width of the superconducting transition, Δ*T*_*C*_. As expected, *T*_*C*_ decreases with decreasing thickness, from ${T}_{C}\simeq 9.1$ K for *d* = 80 nm to ${T}_{C}\simeq 6.5$ K for ≈10 nm. For *d* values lower than ~13 nm, *T*_*C*_ decreases more abruptly, with similar *T*_*C*_ values found for our five thinnest films (Fig. 4). Compared to *T*_*C*_ data reported in the literature by other groups, the *T*_*C*_ of our films is somewhat higher for *d* larger than 17 nm, becoming lower for smaller thicknesses (Fig. 4). This suggests certain level of disorder in our thinnest films. To check that, we examined the width of the superconducting transition, Δ*T*_*C*_, measured as 15÷30 mK, for 25 ≤ *d* <80 nm, increasing to $\simeq 70\div80$ mK for the smallest thicknesses (*d* < 25 nm; Fig. 5). Zhao *et al*.^20^ reported a rapidly rising Δ*T*_*C*_ for *d* < 30 nm, with multiply larger values than those measured in our films with comparable thickness (Fig. 5). These results suggest a superior quality of our superconducting Nb nanofilms at all thicknesses.

What is then the reason for observed lower *T*_*C*_ values for *d* < 17 nm than was the case in ref.^20^? This is likely an unwanted contribution from an oxidized Nb (i.e. NbO_*x*_) layer^26^, formed at the interface between the Nb film and the SiO_2_ substrate, becoming progressively stronger with the reduction of *d*. This oxide layer decreases the quality of the film-substrate interface, and reduces the effective Nb film thickness. Such a contribution is largely reduced on sapphire substrates, which could explain the smoother decrease of *T*_*C*_(*d*) detected by Zhao *et al*.^20^. To verify this, we carried out a specific check, depositing 13.5 nm, 19.5 nm and 22.5 nm thick Nb films on a sapphire substrate (i.e. #*H*14*S*, #*H*1*S* and #*H*11*S*, respectively). Indeed, in those films *T*_*C*_ was significantly higher (~0.5 K of increase of *T*_*C*_ for *d* = 13.5 nm, see yellow filled circles in Fig. 4) than in those deposited on a SiO_2_ substrate, obtained during the same run (#*H*15 & #*H*1; #*H*9: see Table 1). The reduction of *C* and the increase of *l*, in films deposited on sapphire, is in agreement with the hypothesis of a better quality of the layer at the film-substrate interface.

**Figure 6:**
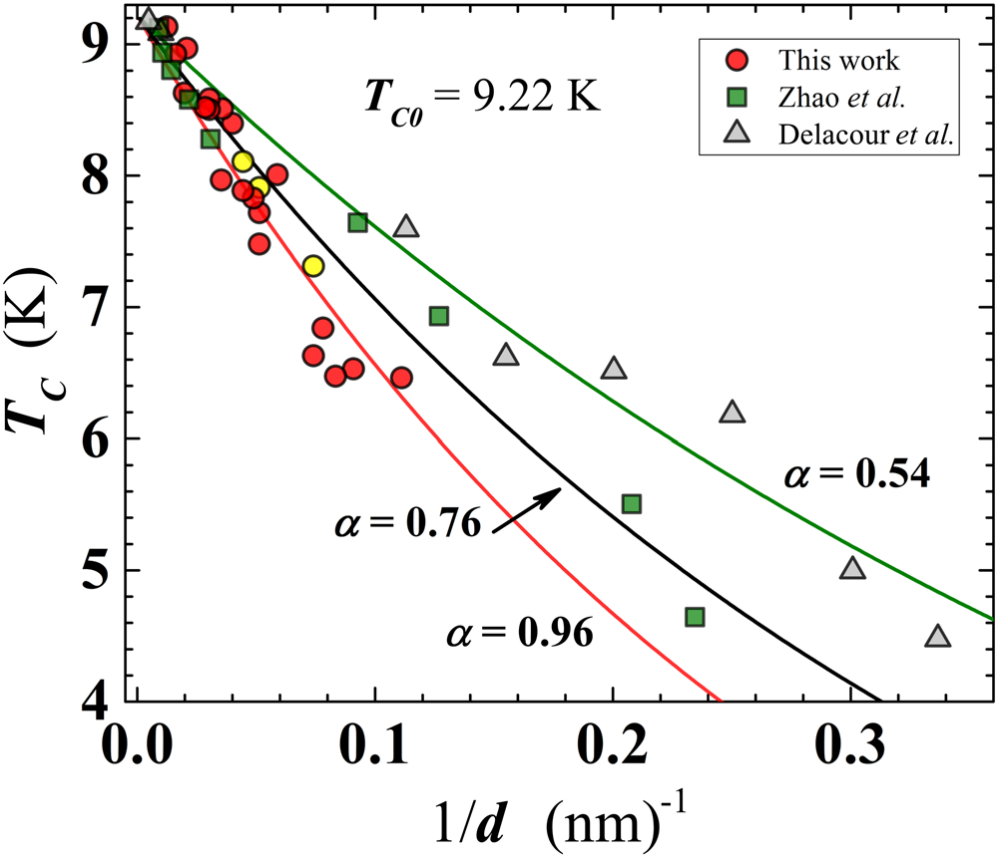
Superconducting transition temperature as a function of the reciprocal film thickness. Present work (SiO_2_ substrate: red filled circles; sapphire substrate: yellow filled circles); Zhao *et al*.^20^ (triangles); Delacour *et al*.^21^ (squares). Continuous lines are least-squares fit, using Eq. (1) (see text), of the different data sets. The effective thickness of the normal layer (in nm) is represented by the corresponding *α* value for each curve.

Another likely effect of the formed NbO_*x*_ layer is that, being a conductive system of electrons in their normal state, it is able to sink Cooper pairs from the superconducting Nb nanofilm, suppressing the condensate fraction and reducing the *T*_*C*_ via proximity effect. This phenomenon should become more effective as the thickness of the Nb nanofilm is reduced so that NbO_*x*_ layer becomes a sizeable fraction of the Nb film thickness. Indeed, the *T*_*C*_(*d*) curve of Fig. 4 shows a smooth decrease of *T*_*C*_ for intermediate-to-large film thickness (i.e. for $20\lesssim d\lesssim 80$ nm), which accelerates for smaller *d* values (i.e. at $d\lesssim 20$ nm). The *T*_*C*_ suppression law, due to the proximity effect, has been derived by McMillan^27^ and is given by:

1 $${T}_{C}={T}_{C0}{(\frac{3.56{T}_{D}}{{T}_{C0}\pi })}^{-\alpha /d},$$

where *α* = *d*_*N*_*N*_*N*_(0)/*N*_*S*_(0) is an effective thickness of the conductive layer at the interface; *T*_*C*0_ = 9.22 K is the bulk *T*_*C*_ of Nb and *T*_*D*_ = 277 K is the Debye temperature. The quantities *N*_*N*_(0) and *N*_*S*_(0) are the density of states in the normal (*N*) and superconducting (*S*) layers, respectively. For simplicity, considering the ratio *N*_*N*_(0)/*N*_*S*_(0) equal to unity, one obtains *α* ≅ *d*_*N*_. Delacour *et al*.^21^ applied Eq. (1) to fit their data set for *T*_*C*_(*d*) of Nb films on sapphire, finding a very small effective thickness of the normal layer of *d*_*N*_ = 0.54 ± 0.02 nm. Following the same procedure and interpretation of Delacour *et al*. we obtain a satisfactory fit of our data for *T*_*C*_(*d*) using Eq. (1) and *d*_*N*_ = 0.96 ± 0.04 nm (Fig. 6). In addition, we have found an intermediate value of *d*_*N*_=0.76 ± 0.03 nm for the *T*_*C*_(*d*) data set of Zhao *et al*.^20^ for films also on sapphire. We conclude that the obtained thickness of NbO_*x*_ in our case (~1 nm) is reasonable, being comparable but larger than found in films grown on sapphire. This analysis confirms the important role played by the substrate. In particular, the absence of reactive oxygen, especially onto the substrate surface, is a mandatory requirement to deposit ultra-thin and ultra-clean Nb films, avoiding or drastically limiting the formation of a metallic oxide at the film-substrate interface.

**Figure 7:**
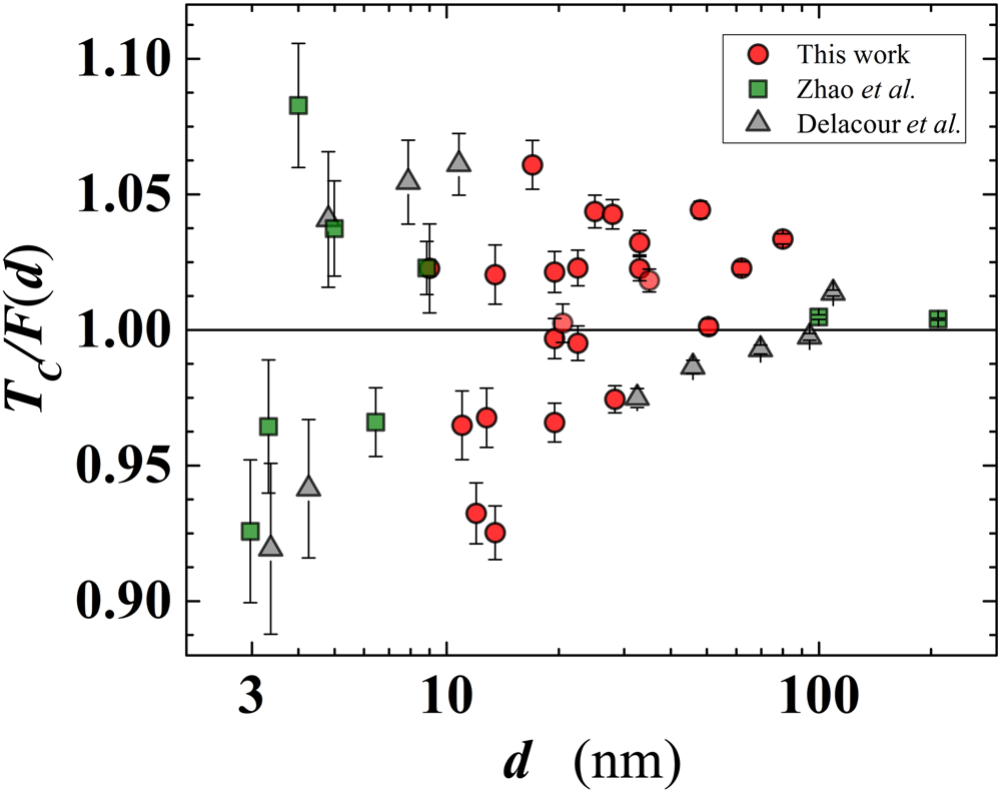
Plot of the ratio *T*_*C*_/*F*(*d*) (see text) as a function of the film thickness for several Nb data sets. The fitting function, *F*(*d*), has been calculated using the theoretically expected value of the Nb bulk transition temperature (i.e. *T*_*C*0_ = 9.22 K). Circles: present work; triangles: Zhao *et al*.^20^; squares: Delacour *et al*.^21^. The error bars are defined by Eq. (S7) of the supplementary information.

At this point it is worth noting that in the presence of an overall suppression of *T*_*C*_(*d*) due to proximity effect, the fitting function given by Eq. (1) can be subtracted from the general data set in order to amplify the visibility of the remaining oscillations of *T*_*C*_ for decreasing *d*. Namely, in this range of thicknesses, one expects that *T*_*C*_ starts to display oscillations due to proliferating quantum size effects and shape resonances associated with confinement effects in the perpendicular direction. We have applied the aforementioned subtraction to our *T*_*C*_(*d*) data, but also to the data of Delacour *et al*.^21^ and Zhao *et al*.^20^ where such oscillations were not considered. Remarkably, in all cases we detected residual *T*_*C*_ oscillations of increasing amplitude for decreasing *d* (shown in Fig. 7). The observed increase of oscillations could be attributed to an incipient effect of the shape resonances when *d* < 20 nm. Namely, the detected oscillations of *T*_*C*_ with amplitude of 5% in the thinnest films are very much comparable to the theoretical predictions for Al and Pb films of similar thickness, discussed in ref.^28^ (and references therein), suggesting their quantum-confinement origin in our Nb nanofilms. As should be the case, for larger thicknesses (*d* > 20 nm) the *T*_*C*_ oscillations in our films get progressively reduced in amplitude.

**Figure 8:**
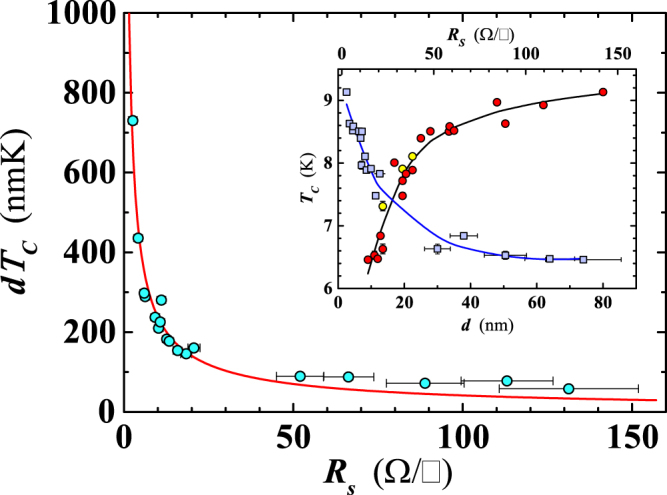
Dependence of the product of the film thickness, *d*, and the superconducting transition temperature, *T*_*C*_, on the sheet resistance (*R*_*s*_) for the investigated Nb nanofilms. The red line is the least-squares fit of the data obtained using Eq. (2). Inset reproduces the behavior of *T*_*C*_ (circles) and of *R*_*S*_ (squares) as a function of the thickness. Yellow filled symbols refer to samples deposited on sapphire substrates. Blue and black lines are guides for the eyes.

As the final characterization of the critical temperature in our films, we discuss the recent findings of Ivry *et al*.^29^ that *T*_*C*_ should exhibit an intricate dependence not only on the film thickness but also on the sheet resistance, *R*_*S*_. Namely, Ivry *et al*. demonstrated a universal relationship among *T*_*C*_, *d* and *R*_*S*_, for films with thicknesses in a broad range (i.e. ≈1 ÷ 10^3^ nm) and belonging to different classes of superconducting materials^29^, showing that experimental data can be well described by the relation:

2 $$d\cdot {T}_{C}=A{{R}_{S}}^{-B},$$

where *A* and *B* are fitting parameters, hereafter considered unitless. We have therefore investigated the behavior of *T*_*C*_ in our films as a function of *R*_*S*_, to test the validity of Eq. (2). Figure 8 shows the very successful fit, yielding *A* = 1350 ± 120 and *B* = 0.76 ± 0.05. Our *B* value is in excellent agreement with the result of Ivry *et al*. for Nb films, while our parameter *A* is larger^29,30^. This fact, in addition to the better quality of our nanofilms, is also due to the different range of data points used for the fit compared to those considered in ref.^29^. The inset of Fig. 8 shows that in our case *T*_*C*_ steeply decreases for $\mathrm{0 < }{R}_{S}\lesssim 20$ ${\rm{\Omega }}/\square$, and then attains an almost constant value for *R*_*S*_ > 50 ${\rm{\Omega }}/\square$.

### Other critical quantities and characteristic length scales of the superconducting state

**Figure 9:**
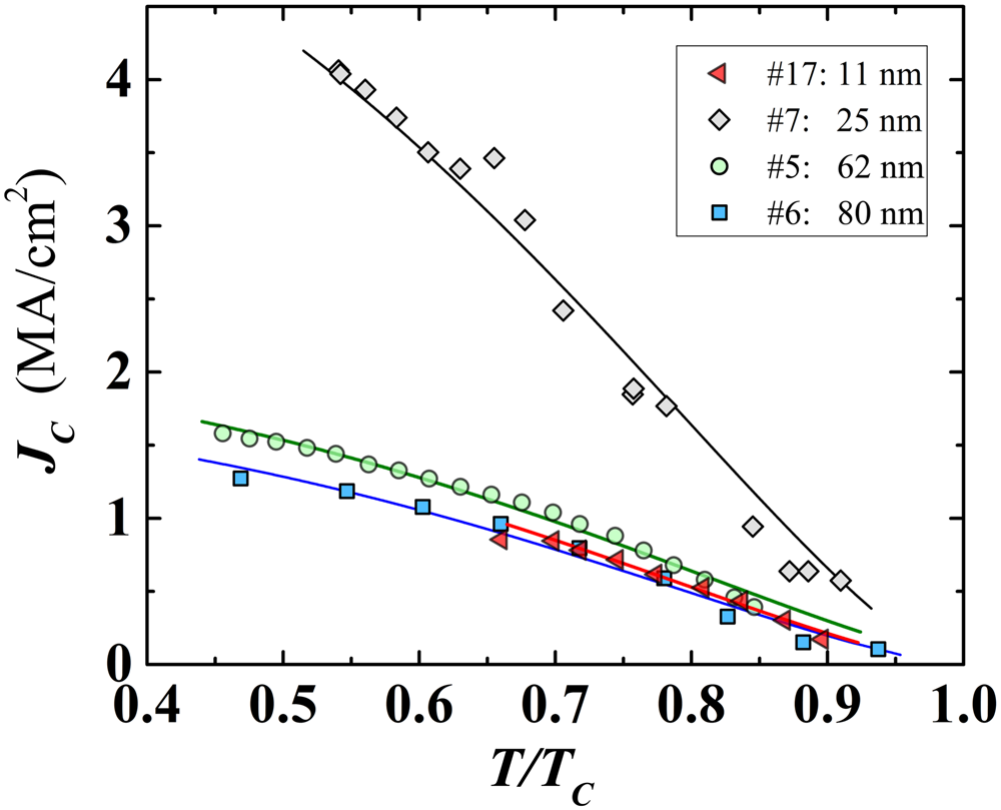
Normalised temperature dependence of the critical current density. Plotted curves refer to four films covering the whole range of thicknesses of the considered Nb nanofilms. Lines are the least-squares fit by the Ginzburg-Landau relation (see the equation S1 in the supplementary material) to the experimental data points.

Additional information about the physical properties exhibited by the here investigated Nb films can be extracted from the behavior of the critical current density, *J*_*C*_, and the maximal magnetic field the superconducting samples can sustain, i.e. the upper critical magnetic field *H*_*C*2_. We measured the critical current at different fixed temperatures and in absence of any applied magnetic field. We assumed as critical current the lowest *I* value causing the jump into the normal state in the I-V curve. The uncertainty in the critical current values was less than 1% (see the supplementary information and Figure S2). The aim is to obtain the functional temperature dependence of these quantities and then extrapolate their values at zero temperature. The dependence of the critical current density, *J*_*C*_, on reduced temperature, *t* = *T*/*T*_*C*_, for selected films (representative of all investigated ones), is shown in Fig. 9. To model the measured *J*_*C*_(*t*) behaviour, we rely on the Ginzburg-Landau (GL) theory, although it is formally valid only near *T*_*C*_. However, to recover the experimentally well-known temperature dependence of thermodynamic critical field ${H}_{c}\propto (1-{t}^{2})$ (as opposed to *H*_*c*_ ∝ (1 − *t*) in standard GL theory), we employ the empirical modifications proposed by Ginzburg^31^ (also corresponding to the temperature dependence from a two-fluid model), as already successfully used in theoretical descriptions of single-crystalline superconductors even far below *T*_*C*_^32^. As a consequence of these modifications, the GL parameter *κ* = *λ*/*ξ* (where *λ* is the penetration depth and *ξ* the coherence length) becomes temperature dependent *κ*(*T*) = *κ*(0)/(1 + *t*^2^), the upper critical field exhibits dependence *H*_*c*2_ ∝ (1 − *t*^2^)/(1 + *t*^2^), and the depairing current density becomes proportional to (1 − *t*^2^)(1 − *t*^4^)^1/2^. For more details, we refer the reader to supplementary material.

**Figure 10:**
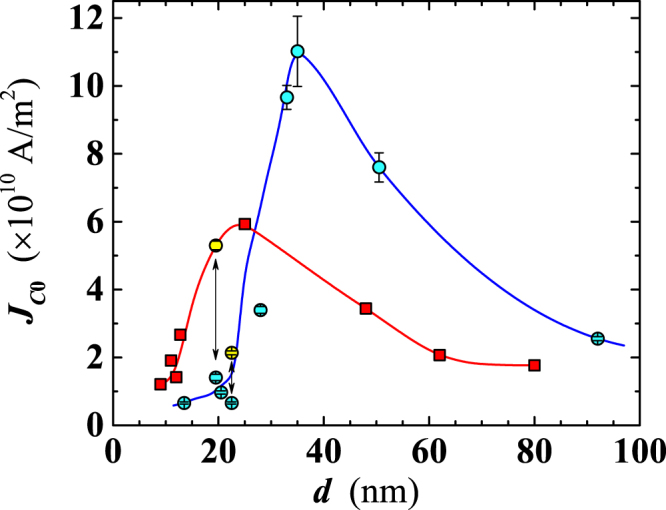
Thickness dependence of the critical current density extrapolated at 0 K. Circles: films *w* = 10 *μ*m wide; yellow filled symbols correspond to the films deposited on sapphire substrates. Squares: *w* = 50 *μ*m wide. The two lines are guides for the eyes.

In absence of an applied magnetic field, the measured critical current density should (nearly) correspond to the depairing current density. Assuming the above modified GL behavior of *J*_*C*_(*T*), we performed the fitting of the obtained data to extract *J*_*C*0_ for films of different thicknesses, having a lateral width of either *w* = 10 *μ*m or *w* = 50 *μ*m. We found a strong dependence of *J*_*C*0_ on both the thickness and the width. It turns out that, compared to the Nb bulk *J*_*C*0_ value of 1.96 MA/cm^2^, our films exhibit considerably higher *J*_*C*0_ for thickness in the range 13 < *d* < 60 nm, with a peak value ~3 and ~5.5 times higher for sample width *w* = 50 *μ*m and *w* = 10 *μ*m, respectively (see Fig. 10). The sizeable increase of *J*_*C*0_ upon narrowing the film width is related to the edge barrier effects for vortex entry, as observed also by Il’in *et al*.^33^. Effects of the film geometry on *J*_*C*_ are expected when the penetration depth becomes comparable to any dimension of film cross-section (*d* or *w*), at the crossover between depinning and depairing mechanism for the critical current. In addition, the increase of *J*_*C*0_ in nanofilms deposited on sapphire evidences once more the important role played by this substrate in improving the superconducting properties of the nanofilms.

**Figure 11:**
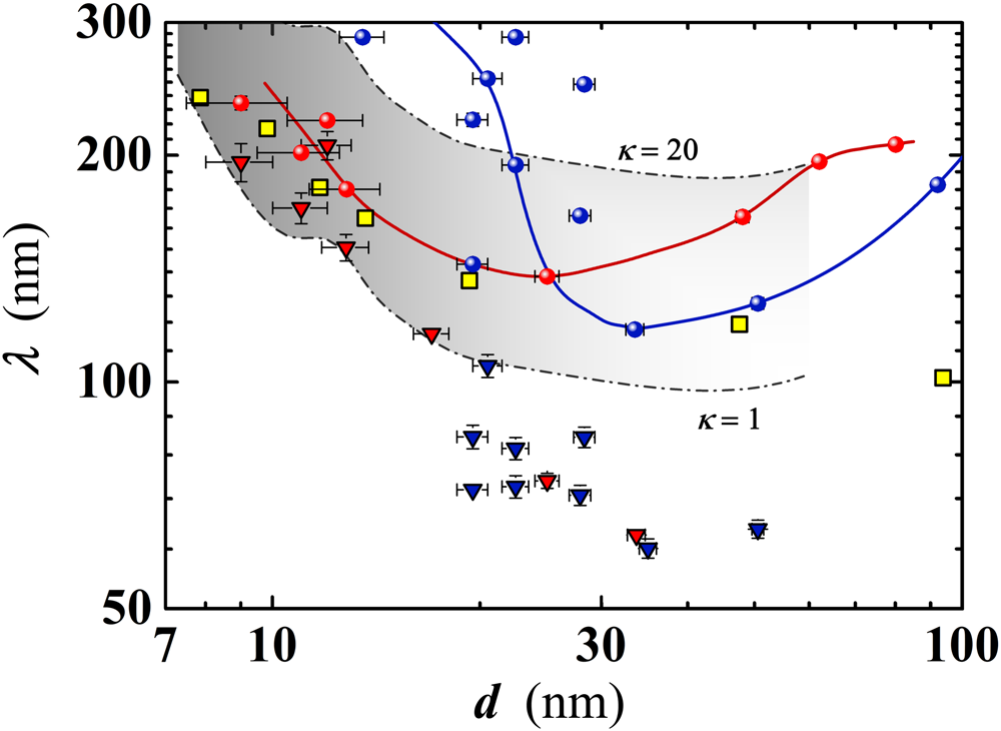
Penetration depth as a function of the Nb film thickness. Values have been calculated by using both Eq. (3), with *κ* = 2.2 (spheres), and Eq. (4) valid in the dirty limit (triangles). Shown data refer to films having width *w* = 10 *μ*m (blue) or *w* = 50 *μ*m (red). Squares: values measured by Gubin *et al*.^22^. Dash and dotted lines delimit the range of *λ* variation (for the case *w* = 50 *μ*m), for *κ* varied between 1 (bottom line) and 20 (top line) in Eq. (3). Continuous lines are guides for the eyes.

The measured critical current densities allow to estimate the penetration depth *λ* of the Nb nanofilms as a function of *d*. For type-II superconductors with thickness smaller than *λ* (2*d* < *λ*), holds the relation of Talantsev and Tallon^34^:

3 $${J}_{C}^{II}(sf)=\frac{{H}_{C1}}{\lambda }=\frac{{{\rm{\Phi }}}_{0}}{4\pi {\mu }_{0}{\lambda }^{3}}(\mathrm{ln}\,\kappa +0.5),$$

where Φ_0_ is the magnetic flux quantum; *μ*_0_ the vacuum permeability and *κ* = *λ*/*ξ* generally assumed to be ≈1 for Nb in the clean limit. ${J}_{C}^{II}(sf)$ represents the current density which generates the self field that induces vortices and itself limits the maximal current in absence of any applied magnetic field. Therefore, under the above mentioned condition, the critical current density is determined only by the fundamental lengths of the superconducting state *λ* and *ξ*, and is independent of geometry and detailed pinning microstructure. Applying Eq. (3) to the measured values of the critical current density we have extrapolated the values of *λ* at the lowest accessible *T* by the experimental setup (≈4 K). The resulting thickness dependence of *λ* is shown in Fig. 11 together with the *λ* values measured in Nb films by Gubin *et al*., using a resonance technique^22^. The agreement between our data and those of ref.^22^ is very good up to *d* ≈25 nm, with an increasing discrepancy for *d* ≈ 50 nm. An alternative check of the thickness dependence of *λ* has been done by using the experimental mean free path *l* (Fig. 3) and the relation valid in the dirty limit^35^:

4 $$\lambda =0.62{\lambda }_{L}\sqrt{(\frac{{\xi }_{0}}{l})},$$

where *λ*_*L*_ = 39 nm and *ξ*_0_ = 38 nm are the Nb bulk value of the London penetration depth and the BCS coherence length, respectively. For thinner films ($d\lesssim 20$ nm) the values of *λ* calculated either by Eq. (4) or Eq. (3) are comparable while diverging, in values and behaviour for thicker films. While *λ* calculated by Eq. (4) preserves the likely correct *d* dependence and decreases towards the bulk limit, its values are more than halved with respect to those obtained in ref.^22^. The apparent disagreement in our *λ*(*d*) data calculated by the two methods, can be accounted for considering that Eq. (3) is valid for $2d\ll \lambda$^34^, condition that appears to be fulfilled in our case only below *d* ≈ 20 nm (Fig. 11). Finally, it is worthwhile to consider also the dependence of *λ* on the value of *κ* in Eq. (3), considering that much larger values of the GL parameter are expected in dirty films compared to single-crystalline samples. Figure 11 reproduces also the possible range of values taken by *λ* for 1 ≤ *κ* ≤ 20 in the thickness range 10 < *d* < 50 nm. Looking at Fig. 11 it is evident that assuming *κ* ≈ 1 (as was actually done in ref.^34^) a better agreement between the two calculation methods of *λ* would have been found.

**Figure 12:**
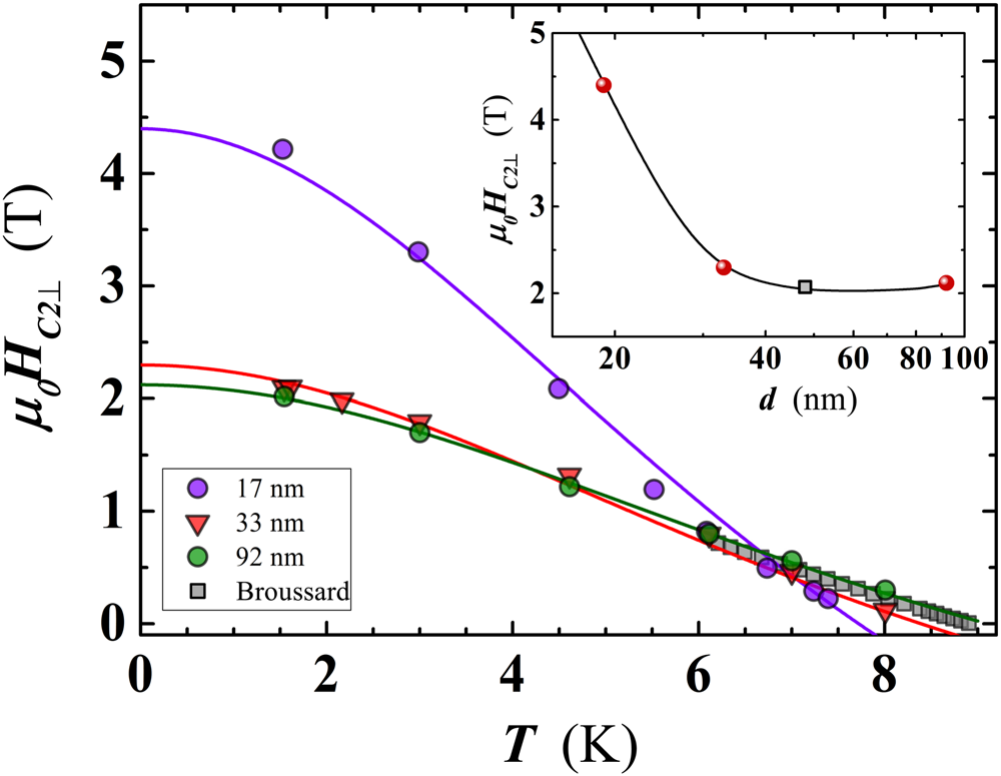
Temperature dependence of the perpendicular critical magnetic field (*H*_*C*2⊥_). For each temperature, the corresponding *H*_*C*2⊥_ value has been derived as the mean value of the applied magnetic fields corresponding to 10% and 90% of the resistivity value at the saturation point (see Figure S3 in supplementary material). Lines are the least-squares fit of the data points by Eq. (5). For comparison, data of Broussard^38^ for the *H*_*C*2⊥_ of a 48 nm thick Nb film have been added to the plot. Inset: thickness dependence of ${H}_{C2\perp }$ at 1.6 K, for the three investigated Nb films (spheres) and for the sample of Broussard^38^ (square) whose value, at 1.6 K, has been extrapolated by Eq. (5) from the experimental data available down to ≈6 K. The black line serve as a guide for the eyes.

Finally, we have also studied the temperature dependence of the perpendicular critical magnetic field, *H*_*C*2⊥_, for three Nb nanofilms having a thickness of 19.5 nm, 33 nm and 92 nm (#*H*1, #*H*8 and #*H*12, respectively). The *H*_*C*2⊥_ value has been determined by measuring the resistivity as a function of the applied magnetic field, at different fixed *T* (Figure S3 in supplementary material). The experimental *H*_*C*2⊥_(*T*) curves for the three films are reproduced in Fig. 12. At a fixed *T*, the *H*_*C*2⊥_(*T*) has been determined as the average of the two *H* values corresponding to the 10% and the 90% of the *ρ*(*H*) saturation value. The two thicker films (i.e. #*H*8 & #*H*12) exhibit practically overlapped curves. Approaching the lowest *T*, *H*_*C*2⊥_(*T*) tends to saturate while a small deviation from the linearity has been detected for the thinner film (i.e. #*H*1) at *T* > 5.5 K. This fact suggests inhomogeneity effects in the film thickness and/or presence of defects due to the reduced *d* value, that is likely related to an interaction of the Nb film with the SiO_2_ of the substrate, as mentioned above in the discussion of the rapid decrease of *T*_*C*_ in that limit. The *T* dependence of *H*_*C*2⊥_ has been captured by a least-squares fit of the experimental values using the relation^36^:

5 $${H}_{C2\perp }(T)={H}_{C20\perp }\frac{[1-{(T/{T}_{C})}^{2}]}{[1+{(T/{T}_{C})}^{2}]},$$

with ${H}_{C20\perp }$ being the value of the orthogonal upper critical magnetic field at zero temperature.

The values of ${H}_{C2\perp }(T)$ allow us to calculate the in-plane coherence length $\xi (T)$ of our films, extracted by the standard GL relation^36^:

6 $${H}_{C2\perp }(T)=\frac{{{\rm{\Phi }}}_{0}}{2\pi {\xi }^{2}(T)},$$

The Ginzburg-Landau coherence lengths at *T* = 0 K, $\xi \mathrm{(0)}$, for the three samples considered in Fig. 12, have been derived using the Eq. (6) and the *H*_*C*20⊥_ values extracted by the Eq. (5). *ξ*(0) raises from $\simeq 7.8\,{\rm{nm}}$ for *d* = 19.5 nm to ≈11.5 nm for *d* ≥ 90 nm (see Table 1), compared to the Nb bulk value of *ξ*(0) = 0.74 *ξ*_0_ ≈ 28 nm.

Our results are in agreement with those reported by Trezza *et al*. for Nb films of comparable thickness^37^, and with values measured by Broussard in a 48 nm thick Nb film^38^. Figure 12 reproduces the ${H}_{C2\perp }(T)$ data of ref.^38^ and shows an overlapping trend with data for our two thicker films, suggesting a saturation of ${H}_{C2\perp }$ for Nb films with *d* larger than 33 nm. The hypothesis has been confirmed also by the ${H}_{C2\perp }(T)$ value of the Broussard film at 1.6 K obtained through a best fit with Eq. (5) (see the inset of Fig. 12).

**Figure 13:**
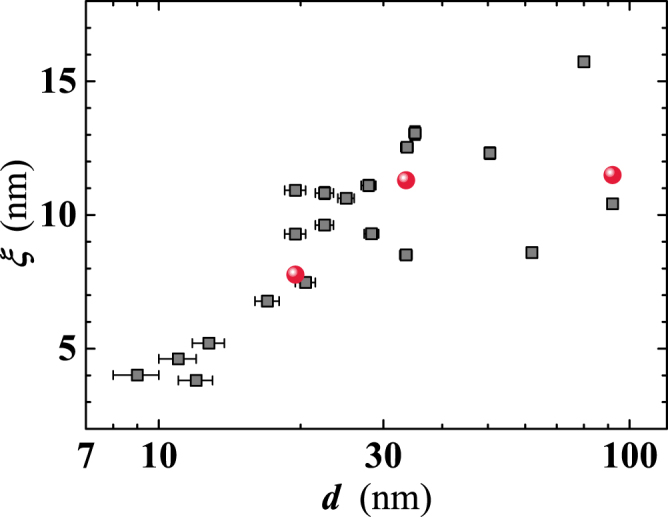
Thickness dependence of the coherence length at zero temperature for investigated Nb films. Squares: values derived by Eq. (7) taking into account the measured mean free path (*l*). Spheres are the *ξ*(0) values calculated by the measured *H*_*C*2⊥_(*T*) and the fitting procedure described in the text.

Finally, the thickness dependence of the superconducting coherence length in our samples has been evaluated using the experimental *l* values, under the validity of the dirty limit condition:

7 $$\xi \mathrm{(0)}=0.855\sqrt{({\xi }_{0}l)},$$

where *ξ*_0_ = 38 nm is the BCS Nb bulk coherence length. The value of the coherence length at 10 K raises from *ξ* = 4 nm for *d* ≈ 10 nm, up to $\xi \simeq 13$ nm for *d* ≈ 35 nm. For larger thicknesses we observe a saturation effect albeit data points appear somewhat scattered (Fig. 13). In any case, the *ξ* values calculated by Eq. (7) are in very good agreement with those derived from the *H*_*C*2⊥_(*T*) measurements (Fig. 13). Finally, we note that, at *d* = 19.5 nm (#*H*1), $\xi \mathrm{(0)}\simeq 7.8$ nm evidences presence of smaller Cooper pairs, exhibiting shrinking by a factor of five compared to the bulk value.

## Discussion and Conclusions

The comprehensive experimental characterisation of metallic and superconducting properties of Nb nanofilms reported in this work demonstrates two different regimes depending on the thickness. For large thickness, in the range 25 nm < *d* < 80 nm, all measured electrical properties evolve smoothly for decreasing *d*. The Nb nanofilms are found to be of high quality and superconductivity has a 3D character. Around *d* = 25 nm, normal and superconducting state properties display an abrupt change in their behavior. Below 25 nm of thickness, superconductivity becomes progressively 2D, realizing a 3D-2D dimensional crossover. This dimensional crossover for decreasing thickness results to be particularly evident in the sudden suppression of *T*_*C*_, in the peaked behaviour of the critical current density and when comparing the evolution of the penetration depth and the correlation length as a function of thickness. Approaching *d* = 10 nm, novel quantum phenomena start to emerge: (i) on top of the overall drop of *T*_*C*_, for decreasing *d*, remaining oscillations of *T*_*C*_(*d*) indicate incipient quantum size effects. (ii) A factor of five enhancement of the penetration depth for the thinnest films with respect to the bulk has been observed. This points to a strengthening of the type-II magnetic character below 20 nm of thickness. Our measured penetration depth *λ*(*d*) compares in a satisfactory way with other data of *λ*, in the range of thickness *d* ≤ 25 nm. (iii) A sizeable amplification of the upper critical magnetic field is associated with a considerable shrinking of the Cooper pair size. The thinnest Nb nanofilms are close to the BCS-BEC crossover regime, predicted for very thin nanofilms. Close to *d* = 25 nm the Nb nanofilms show optimized superconducting properties: maximal ${J}_{C}\simeq 6$ MA/cm^2^, *λ* = 150 nm, $\xi (0)\simeq 10$ nm, *H*_*C*2⊥_(*T*) = 2 T, *T*_*C*_ = 8 K, and a narrow superconducting transition of ${\rm{\Delta }}{T}_{C}=40$ mK. Therefore, around $d\simeq 25$ nm of thickness, Nb nanofilms constitute an ideal platform for nanostructuring in the form of stripes for the fabrication of nano superconducting devices. In addition, we have shown that the thickness dependence of the mean free path of the carriers plays a crucial role in understanding the thickness dependence of the superconducting properties, mainly in the penetration depth and in the coherence length. The effects of disorder are therefore entangled with the dimensional crossover and with the BCS-BEC crossover occurring below few tens of nanometers. Finally, we have found that the amplification effects of the superconducting properties reported in this work depend on the substrate and on the lateral width of the nanofilms. Optimal superconducting properties have been demonstrated using sapphire as a substrate and squeezing the lateral size of the nanofilms toward few micrometers, furthermore suggesting great potential in the fabrication of superconducting nanostripes of Nb at the nanometer scale.

## Methods

Niobium nanofilms have been deposited at room temperature on thermally oxidized Si wafer (silicon oxide thickness: 300 ÷ 500 nm), by an ultra high vacuum DC sputtering system, in a base pressure of about 2 × 10^−9^ mbar. Film thickness has been varied from about 9 nm to 90 nm, keeping constant the deposition rate at 0.65 nm/s (see Table 1). Scanning electron microscopy (SEM) analysis has been carried out on some films by a FEI Quanta^*TM*^ 3D FIB (Nanofacility Piemonte, INRIM).

For characterization of the electrical properties samples have been shaped in a Hall bar geometry, 1 ÷ 2 cm long, 10 *μ*m and 50 *μ*m wide. The resistivity, *ρ*(*T*), and the current-voltage (I–V) characteristics have been measured as a function of the temperature, in the range 4 ÷ 300 K, by a He closed cycle cryostat (Advanced Research System mod. 210 DE) equipped with two silicon diode thermometers (Lakeshore DT-670, one of which was calibrated with a maximum error of 6.3 mK) and a temperature controller Lakeshore mod. 332. Resistivity and I-V characteristics have been measured sourcing a constant current (Keithley mod. 220), monitored either by a pico-ammeter (Keithley mod. 6487 for *ρ*(*T*)) or by a multimeter (Hewlett-Packard mod. 34401 A for I-V). The voltage drop has been detected by a multimeter (Keithley mod. 2000). For the measure of *ρ*(*T*) the current sourced has been in the range 1 ÷ 50 *μ*A. Depending on the kind of characterization, measures have been executed either with (e.g. I-V characteristics) or without *T* stabilization (e.g. *ρ*(*T*)). In the former case, a *T* stability better than 50 mK has been achieved below 15 K. In the latter approach, the variation of *T* during data acquisition was lower than 20 ÷ 30 mK. The superconducting transition temperature *T*_*C*_ as well as the width of the transition was measured by a liquid He-cryostat equipped with a silicon calibrated thermometer. For the determination of the upper critical magnetic field, *H*_*C*2_, a liquid ^4^He cryostat, Oxford Instruments Teslatron 16T has been used, equipped with a superconducting magnet (up to 16T) and a variable temperature insert working from 300 K down to 1.5 K. The resistance values were measured by using either a PICOWATT AVS-47 AC or a Lakeshore mod. 370 AC resistance bridge.

## Electronic supplementary material

Supplementary Information
