# Supplementary material for: Dimensional crossover and incipient quantum size effects in superconducting niobium nanofilms
Source: Sci Rep. 2018 Mar 16;8:4710. doi: 10.1038/s41598-018-22983-6 (PMC5856833; doi:10.1038/s41598-018-22983-6)
Supplement: Supplementary file 1 — Supplementary Information [file 41598_2018_22983_MOESM1_ESM.pdf]

Dimensional crossover and incipient quantum size effects in superconducting niobium nanofilms

Nicola Pinto[1,2,*], S. Javad Rezvani[1,3], Andrea Perali[4], Luca Flammia[1,5], Milorad V. Milošević[5], Matteo Fretto[3], Cristina Cassiago[3], and Natascia De Leo[3]

[1]School of Science and Technology, Physics Division, University of Camerino, via Madonna delle Carceri 9, Camerino, 62032, Italy
[2]INFN, Sezione di Perugia, via Pascoli, Perugia, 06123, Italy
[3]Istituto Nazionale di Ricerca Metrologica (INRiM), Strada delle Cacce, 91, Torino, 10135, Italy
[4]School of Pharmacy, Physics Unit, University of Camerino, Camerino, 62032, Italy
[5]Department of Physics, University of Antwerp, Groenenborgerlaan 171, B-2020 Antwerp, Belgium
[*]corresponding author: nicola.pinto@unicam.it

ABSTRACT

1 Methods

1.1 Experiment

For electrical and magnetic characterisation of the Nb films, a typical Hall bar geometry has been fabricated. Niobium has been used as material for the fabrication of both pads and lateral arms (for the voltage probe) (Figure S1).

1.2 Superconducting critical current density

The superconducting critical current density at zero T, J_{C0}, has been extracted by the current-voltage characteristic carried out sourcing slow-sweep currents at fixed T, in the range from 4 K to T_C. Hysteretic I-V curves have been measured for the investigated films, similar to that shown in Figure S2. Because of the sharp jump in the I-V curves, we assumed as critical current density at a given temperature, $J_C(T)$, the J value corresponding to the jump into the normal state in the I-V curve (Figure S2). These values have been determined with an uncertainty $\lesssim 1\%$. The value of J_{C0} has been derived by a least squares fit of the $J_C(T)$ curve assuming the mean-field behavior of $J_C(T)$ predicted by the Ginzburg-Landau (GL) theory for depairing critical current density:

$$J_C(T) = J_{C0}\left[1-(T/T_C)^2\right]^{3/2}\left[1+(T/T_C)^2\right]^{1/2}. \tag{S1}$$

We experimentally check this T dependence of the $J_C(T)$ (Eq. S1) for several Nb layers covering the range of thicknesses and the two Hall bar widths (10 μm and 50 μm) used in the present study.

1.3 Error analysis of T_c oscillations

According to the McMillan model, the T_C suppression law is related to the thickness d by the following relation

$$T_C(d) = T_{C0}\left(\frac{3.56\times T_D}{\pi\times T_{C0}}\right)^{-\frac{\alpha}{d}} = T_{C0}\exp\left\{-\ln\left(\frac{3.56\times T_D}{\pi\times T_{C0}}\right)\frac{\alpha}{d}\right\}, \tag{S2}$$

where $T_D = 277$ K is the Debye temperature, and $T_{C0} = 9.22$ K is the Nb bulk critical temperature. The quantity α is the parameter of the model which corresponds to the normal layer thickness d_N when we set the superconducting-to-normal density ratio equal to unity.

The method of least squares is an analytical technique for finding the fitting curve of the model which optimally describes a set of data. Using the McMillan model, the measured values of d and T_C are inserted into the Eq.S2 to determine the optimal parameter α which defines the behaviour of the nonlinear fitting curve $F(d)$. For semplicity we use unit weighting for all data points (from 1 to n) because we cannot infer any estimate of their uncertainties and therefore we assume that every datum point has the same weight. In order to get the parameter α, the procedure for the least squares solution needs to be iterative. For each iteration the solution is obtained with the method described in ref.[1]

Accordingly, here we report the procedure related to the least squares solution for the McMillan model. Starting with an initial guess of $\alpha_{\text{init}} = 1$ and with the set of data (d_i, T_C^i) $(\forall i \in \{1, \ldots, n\})$, we define

$$C = \sum_{i=1}^{n} \left[g_{\alpha}^{i}\right]^{2} \qquad V = \sum_{i=1}^{n} T_C^i g_{\alpha}^{i}, \tag{S3}$$

where $g_{\alpha}^{i} = \frac{\partial T_c(d_i)}{\partial \alpha}$. It is worth noting that in the equation for V we consider values of the dependent variable T_C^i minus the computed value $T_C(d_i)$ obtained with α_{init}.

The parameter α_{calc} is calculated by solving the matrix equation

$$\alpha_{\text{calc}} = C^{-1} V \tag{S4}$$

where calculated value of α_{calc} will no longer be the final solution but it represents the change in the value of the initial guess a_{init}

$$\alpha_{\text{new}} = \alpha_{\text{init}} + \alpha_{\text{calc}} \tag{S5}$$

and the value of α_{new} is then used as initial guess α_{init} for the next iteration. This process is continued until convergence, i.e., when the fractional change is less than a specified value of ε (we use $\varepsilon = 10^{-5}$).

$$\left| \frac{\alpha_{\text{calc}}}{\alpha_{\text{init}}} \right| \leqslant \varepsilon \tag{S6}$$

where Eq.S6 has to be modified if α_{init} is zero or very close to zero and then the condition will depend on the absolute value of α_{calc} and not on the relative value.

When the convergence is achieved and the optimal parameter α_{opt} is determined, the fitting curve $F(d)$ corresponds to the function $T_C(d)$ with α_{opt}. The uncertainty σ_F for every data point i is:

$$\sigma_F^i = \left(\frac{S}{n-1} \left[\frac{\partial F(d_i)}{\partial \alpha_{\text{opt}}} \right]^2 C^{-1} \right)^{1/2} \tag{S7}$$

where the weighted sum of squared residual $S = \sum_{i=1}^{n} \left[T_C^i - F(d_i)\right]^2$.

The ratio $\frac{T_C}{F(d)}$ is a good quantity for detecting the T_C oscillations at low thicknesses. The uncertainty $\sigma\left(\frac{T_C}{F(d)}\right)$ is only determined by σ_F and we get

$$\frac{T_C}{F(d) \pm \sigma_F} = \frac{T_C}{F(d)} \frac{1}{1 \pm \frac{\sigma_F}{F(d)}} \simeq \frac{T_C}{F(d)} \left(1 \pm \frac{\sigma_F}{F(d)}\right) = \frac{T_C}{F(d)} \pm T_C \frac{\sigma_F}{[F(d)]^2} \tag{S8}$$

where the approximation is due to the use of the infinite geometric series for $\|x\| = \| \pm \frac{\sigma_F}{F(d)} \| < 1$ which is truncated at the second term. The uncertainty $\sigma\left(\frac{T_c^i}{F(d_i)}\right)$ for every data point i is therefore:

$$\sigma\left(\frac{T_C^i}{F(d_i)}\right) = T_C^i \frac{\sigma_F^i}{[F(d_i)]^2}. \tag{S9}$$

References

1. Wolberg, J. Data Analysis Using the Method of Least Squares. *Springer-Verlag, Berlin* (2006).

2 Figures

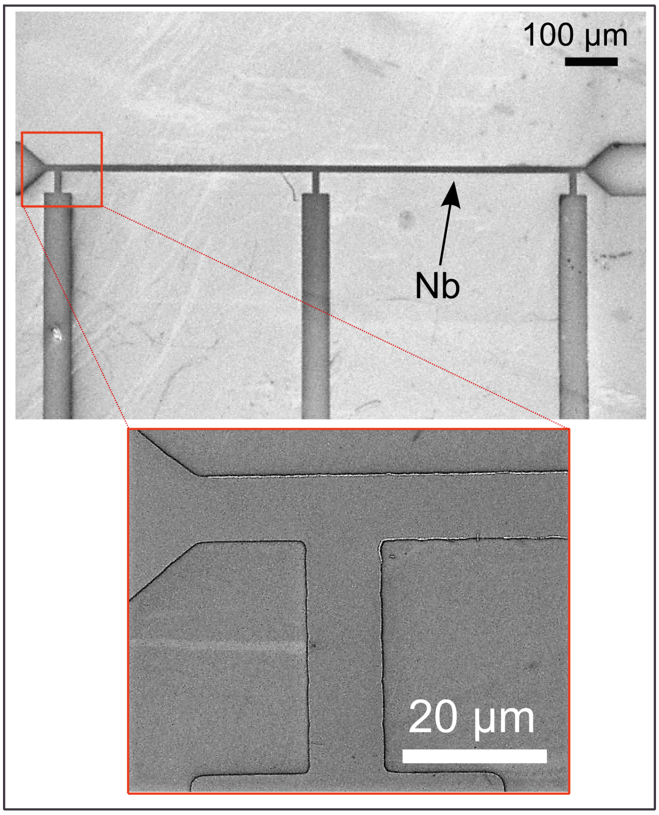

Figure S1. Scanning electron microscopy of a Hall bar device obtained by a 25 nm thick Nb film (#7, see Table I) deposited on SiO_2. Five large Nb contacts, two horizontal (at the midlle of the image) and three vertical are used to source the current and to measure the voltage drop (a couple of them), respectively. Inset: magnification of the contact area zone (red box) showing a uniform and homogenous formation of a Nb layer also at the contact area.

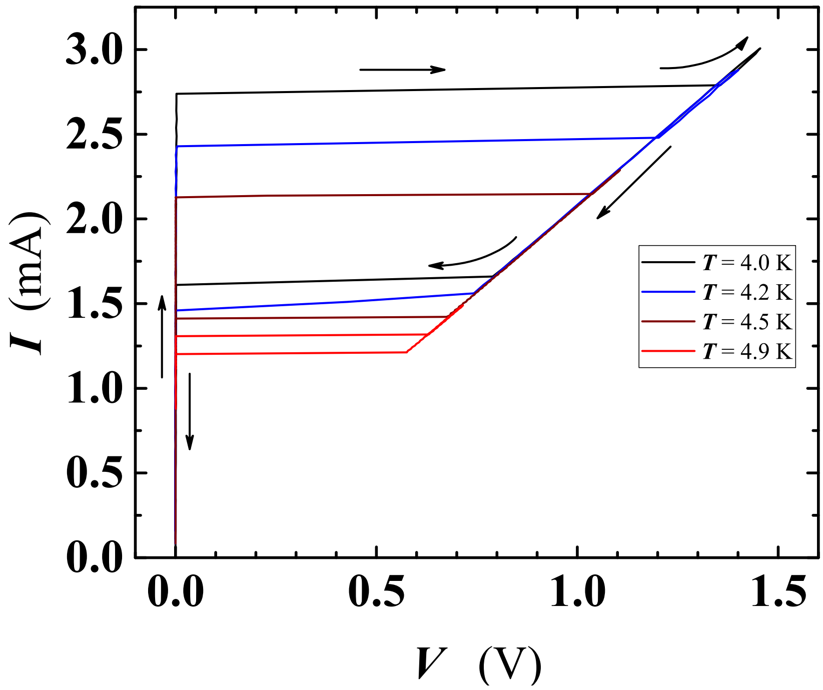

Figure S2. Typical hysteretic current-voltage curves, at few selected T, for the film #12 (see the Table I), used to calculate the superconducting critical current density (see Figure 9). The arrows indicate the sweep direction for the curve measured at 4 K.

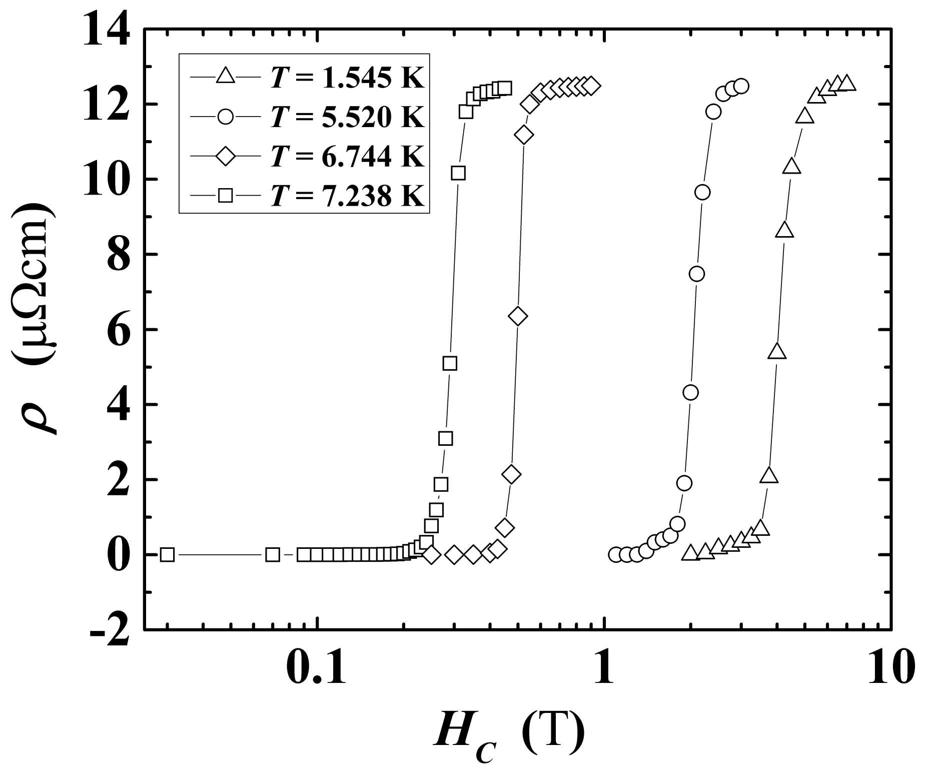

Figure S3. Applied magnetic field dependence of the resistivity, at several temperatures, for a Nb film of 17 nm of thickness (film #*H*1). Lines are a B-spline interpolation of the measured values.
